# Tumor-associated macrophages in gastric cancer peritoneal metastasis: orchestrating immune evasion, niche remodeling, and therapeutic resistance

**DOI:** 10.3389/fimmu.2026.1944126

**Published:** 2026-09-02

**Authors:** Min Chen, Yuyang Li, Huinan Wang, Xuyang Li, Qian Shen, Lei Yang

**Affiliations:** 1 Medical School of Nantong University, Affiliated Tumor Hospital of Nantong University, Nantong, China; 2 Department of Hematology and Lymphoma, Affiliated Tumor Hospital of Nantong University, Nantong, China; 3 Department of Oncology, Affiliated Tumor Hospital of Nantong University, Nantong, China

**Keywords:** gastric cancer, immunosuppressive microenvironment, macrophage polarization, peritoneal metastasis, tumor-associated macrophages

## Abstract

Peritoneal metastasis is the most devastating form of progression of gastric cancer, characterized by strong immune suppressive properties and resistance to treatment. Tumor-associated macrophages (TAMs) are the main population of immune cells in the peritoneal cavity and participate in nearly every step of this process. In this Review, we focus on the two major origins of peritoneal macrophages (embryonic-derived GATA6+ resident cells and monocyte-derived CCR2+ cells), and we describe how gastric cancer cells exploit their plasticity by metabolic signals, exosomal oncomiRs, paracrine factors and so on to push them into a pro-metastatic phenotype. Reprogrammed macrophages proceed to participate in detachment of tumor cells, resistance to anoikis, escaping across mesothelium and stimulating angiogenesis and lymphangiogenesis, but also instigate an immunosuppressive environment suppressing T cell activity and increasing chemoresistance. Important molecular players that we describe here are CCL2/CCR2 and CSF-1/CSF-1R signaling axes, the STAT3/STAT6/PI3K axis and a complement-driven switch of TAMs from a cathepsin (CTS)-high to complement component 1q(C1q)-high state that promotes immune evasion. We also discuss the role of exosome-mediated cross-talk and give an overview of novel therapies against macrophages such as blocking their recruitment, reprogramming their polarization or using chimeric antigen receptor macrophage (CAR-M) therapy, typically in combination with hyperthermic intraperitoneal chemotherapy (HIPEC) or immune checkpoint inhibitors. Bringing together the latest advances, we highlight key gaps in our understanding and suggest a direction for dismantling the macrophage-orchestrated metastatic niche with the ultimate goal of improving outcomes of this devastating disease.

## 1 Introduction

Gastric cancer is the fifth most common cancer in the world and the fifth leading cause of death (1). One of the most serious complications is peritoneal metastasis, and the mortality rate is extremely high. At the initial diagnosis, about 14% of patients showed signs of peritoneal metastasis. Globally, among patients undergoing curative-intent surgery for advanced disease, up to 50% of people will have peritoneal recurrence after surgery (2). Once a recurrence occurs, the median survival will be shortened to only 3 to 6 months (3).

Treatment outcomes remain poor, mainly because the peritoneal cavity forms an immunosuppressive environment, which will hinder the treatment. For example, systemic chemotherapy is limited by the peritoneal barrier - the concentration of drugs in the peritoneal cavity is much lower than that in the bloodstream. Molecular targeted drugs and immunotherapy perform well in a variety of solid tumors, but have little effect on the foci of gastric cancer metastasis to the peritoneum. This failure shows the immunosuppressive characteristics of the metastatic microenvironment (4).

Macrophages are the largest number of immune cells in the abdominal cavity and play a central role in the tumor immune microenvironment (TIME) during peritoneal metastasis. They are mainly derived from two sources: one is from the resident cell group of embryonic precursors, and the other is recruited from monocytes in the blood (5). Under normal circumstances, these macrophages will actively identify threats and participate in tissue repair. However, once the tumor begins to form, gastric cancer cells will induce these macrophages to change their behavior patterns and turn to an M2-like (alternatively activated) tumor-promoting phenotype, which will eventually promote the growth of cancer (6).

In gastric cancer tissue, tumor-associated macrophages (TAMs) obviously change to M2 phenotype. These cells promote the formation of new blood vessels by releasing vascular endothelial growth factor (VEGF), interleukin-1β (IL-1β), and transforming growth factor-β1 (TGF-β1) (7), and make tumors escape immune attacks through multiple ways (8). With the progress of single-cell and spatial transcriptomics technologies, people have been able to isolate different macrophage subgroups and draw a close interaction map between them and gastric cancer cells. At present, many new TAM subtypes have been found. For example, Secreted phosphoprotein 1 (SPP1)+ TAMs can promote angiogenesis and inhibit immune activity (9), complement component 1q (C1q)+TAMs use complement signals to help tumors escape immune clearance (10), and CCL18 highly expressed TAMs promote the migration and invasion of tumor cells (11). These subtypes appear to exert distinct tumor-promoting functions, in part by modulating immune metabolites and facilitating immune evasion, which may also influence the efficacy of immune checkpoint inhibitors.

This review shows the current research progress on how macrophages promote peritoneal metastasis of gastric cancer. We discussed the origin of these cells, phenotypic plasticity, and their role at various stages of the metastatic process, and introduced the relevant key signaling pathways. In addition, it also covers the treatment strategies for macrophages. Finally, the review outlines the main challenges that still exist and points out several particularly optimistic future development directions.

## 2 Origin, polarization and functional remodeling of macrophages

### 2.1 Cellular origins of macrophages

Macrophages in the peritoneal metastasis of gastric cancer mainly come from two main lineages: tissue resident cells and cells derived from monocytes (12). Tissue resident macrophages originate from embryonic precursor cells in the yolk sac or fetal liver. They can be maintained autonomously in adulthood without relying on the supplementation of bone marrow monocytes. In the abdominal cavity, these cells significantly express transcription factors GATA6, as well as surface markers such as TIM4 and ICAM2. Most of them are concentrated in the milky spots of the greater omentum (13), which is the main attachment site for early peritoneal dissemination. Embryonic source resident macrophages expressing GATA6 and TIM4 are the main constructors of the early pre-metastatic microenvironment.

The second genealogy involves circulating monocytes. As the tumor progresses, monocytes in the peripheral blood are recruited into the peritoneal cavity by relying on the chemotaxis of CCR2. Once arrived, local environmental signals will cause them to differentiate into macrophages (14). The subgroup showed low expression levels of GATA6, while higher levels of CCR2 and CD226 (15). With the development of tumors, the number of monocyte-derived macrophages gradually increases, especially in patients with late malignant ascites.

Macrophages from these two sources are also different in terms of phenotypic flexibility. In the past, it was generally believed that macrophages could easily switch between M1 and M2 phenotypes. However, more and more evidence shows that macrophages of embryonic and monocyte origin mostly maintain their own functional state, and the mutual conversion between the two types of polarization is far less common than imagined by earlier models.

### 2.2 Macrophage polarization

Macrophage polarization means that it can adjust its own function according to the signals of the local tissue environment, thus showing different phenotypes. The traditional classification divides it into two categories: classically activated M1 macrophages and alternatively activated M2 macrophages (16). M1 polarization is mediated by IFN-γ and LPS. These cells express high levels of inducible nitric oxide synthase (iNOS) and major histocompatibility complex II (MHC II), and release a series of pro-inflammatory cytokines - such as IL-1, IL-6, IL-12 and TNF-α, thus giving it anti-tumor activity. In contrast, M2 macrophages are activated by IL-4, IL-13, IL-10 and TGF-β, which are characterized by elevated levels of CD163, CD206 and arginase 1 (Arg1) (17, 18). Their main functions focus on immunosuppression, tissue repair, angiogenesis and tumor growth regulation.

As the core cells of the innate immune system, macrophages exhibit remarkable functional plasticity. The classical M1/M2 dichotomy remains a useful conceptual framework for interpreting *in vitro* polarization driven by defined stimuli and for simplifying macrophage states in early studies. However, *in vivo*, especially in the chronically inflamed and hypoxic peritoneal metastatic microenvironment, this binary model is insufficient (17, 19): macrophages are not locked into terminal M1 or M2 states but instead exist on a continuous, context-dependent polarization gradient. Recent single-cell RNA sequencing studies of malignant ascites from gastric cancer patients have directly challenged the rigid M1/M2 dichotomy by identifying multiple macrophage subtypes, including CTS-high and C1q-high TAMs, that can simultaneously express inflammatory and pro-tumor genes (10, 20). These subtypes, whose distinct functional programs are discussed in detail in Section 4.3, illustrate the limitations of assigning single M1/M2 labels to TAMs. Moreover, chronic injury-associated macrophages may display an inflammatory, M1-like signature yet still support tumor progression, functionally distinct from classical IFN-γ-polarized anti-tumor M1 macrophages. This continuous multi-subtype spectrum better reflects peritoneal macrophage biology and highlights the plasticity of TAMs in gastric cancer peritoneal metastasis.

### 2.3 Molecular mechanisms underlying pro-tumorigenic macrophage reprogramming by gastric cancer cells

In the tumor microenvironment, cancer cells are not just bystanders, but can actively promote the transformation of TAMs into phenotypes that promote tumor growth (21, 22). This reprogramming process is realized through multiple interrelated regulatory mechanisms, including metabolic reprogramming, exosome-mediated signal exchange and paracrine signals. At the metabolic level, gastric cancer cells reshape the peritoneal environment by ingesting a large amount of glucose and secreting lactic acid. As a by-product of tumor glycolysis, lactic acid can guide macrophages into the M2 state through the action of the mammalian target of rapamycin (mTOR)/hypoxia-inducible factor 1α (HIF-1α) pathway (23, 24). Under the highly hypoxic conditions characteristic of the gastric cancer tumor microenvironment (TME), lactate-induced mTOR activation stabilizes HIF-1α, which then directly binds to hypoxia-response elements in the promoters of M2-associated genes such as Arg1, CD206, and IL-10, while simultaneously suppressing M1-related genes, thereby shifting macrophages toward an M2-polarized pro-tumor phenotype (25). Moreover, lactate serves as a substrate for histone lactylation, an emerging epigenetic modification that regulates gene expression in macrophages. Lactate-induced histone lactylation promotes the transcription of pro-tumor and M2-associated genes in TAMs, thereby contributing to the formation of the pre-metastatic niche and facilitating peritoneal metastasis (26, 27). In addition, the fatty acids released by cancer cells can also upregulate the expression of CD36 on TAMs, and this signal of dependence on CD36 further promotes the polarization of TAMs to M2 phenotype (28, 29).

Communication mediated by the exosome provides another way. Under hypoxic conditions, cancer cells will package tiny RNAs such as miR-21-3p into the exosome, and the process is regulated by HIF-1α and HIF-2α. When macrophages ingest these vesicles, the signals produced promote their polarization to M2 and help establish an immunosuppressive environment (30, 31). The role of this mechanism in gastric cancer is receiving more and more attention. A recent study discovered a new mechanism: the expression of METTL3 in gastric cancer cells increased, resulting in the enhancement of m6A methylation, thus promoting the generation of exosome. The released exosome carries miR-17–92 clusters, which can target SRCIN1 in macrophages, activate the SRC signaling pathway, and induce the release of immunosuppressive cytokines, effectively constructing a pre-metastatic microenvironment (32).

**Figure 1:**
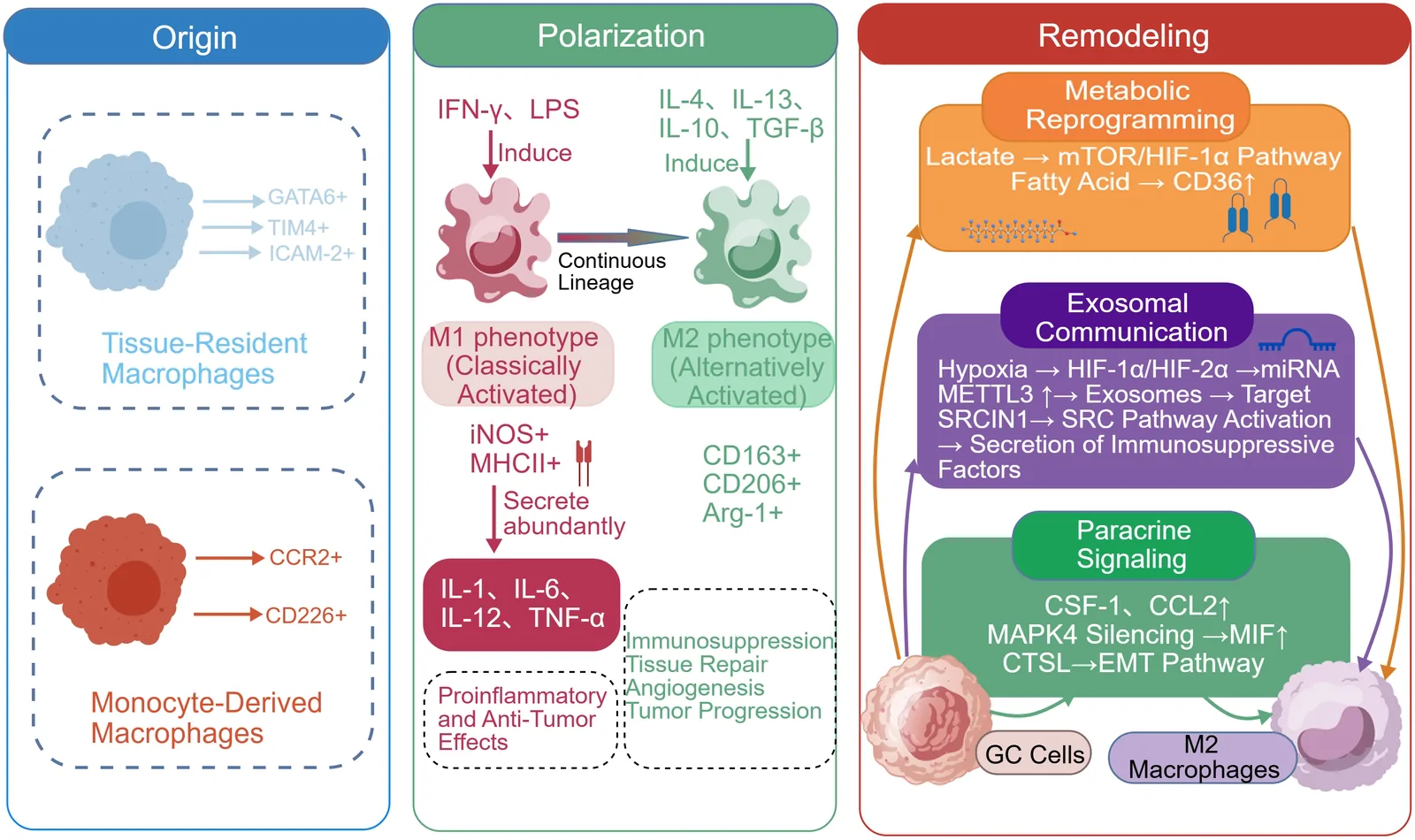
Schematic overview of macrophage origins, polarization, and functional remodeling in the gastric cancer peritoneal metastatic microenvironment. Tissue-resident macrophages (GATA6^+^ TIM4^+^ ICAM-2^+^) originate from embryonic precursors, whereas monocyte-derived macrophages (CCR2^+^ CD226^+^) are recruited from peripheral blood. Polarization toward an M1 phenotype (iNOS^+^ MHCII^+^) is induced by IFN-γ and LPS, producing proinflammatory cytokines (IL-1, IL-6, IL-12, TNF-α) with anti-tumor effects; M2 polarization is driven by tumor-derived signals and promotes immunosuppression and tumor progression. Functional remodeling of tumor-associated macrophages (TAMs) involves: metabolic reprogramming (lactate activates mTOR/HIF-1α; fatty acids upregulate CD36); exosomal communication (hypoxia-induced HIF-1α/HIF-2α regulate miRNAs, and METTL3-enriched exosomes carrying miR-17-92 cluster target SRCIN1 to activate SRC signaling, enhancing immunosuppressive factor secretion); and paracrine signaling (CSF-1, CCL2, MIF, and CTSL/EMT pathway) that collectively foster M2 polarization and establish a pre-metastatic niche.

The paracrine factor constitutes another level of regulation. Gastric cancer cells can secrete two chemokines, CSF1 and CCL2, which can recruit monocytes into the abdominal cavity and guide them to differentiate into M2-based macrophages (33). For example, after silencing MAPK4 in tumor cells, the secretion of MIF will increase, thus promoting TAM polarization and starting a self-enhancing negative feedback cycle to accelerate metastasis diffusion (34, 35). In addition, CTSL released by macrophages enhances the migration ability and metastasis ability of gastric cancer cells by inducing epithelial-mesenchymal transition (EMT). At the same time, it strengthens the M2 polarization state of macrophages themselves and forms a continuous pathological feedback loop (36). **Figure 1** summarizes these multiple regulatory mechanisms.

## 3 Key steps and interaction networks of macrophage-mediated regulation in peritoneal metastasis

**Figure 2:**
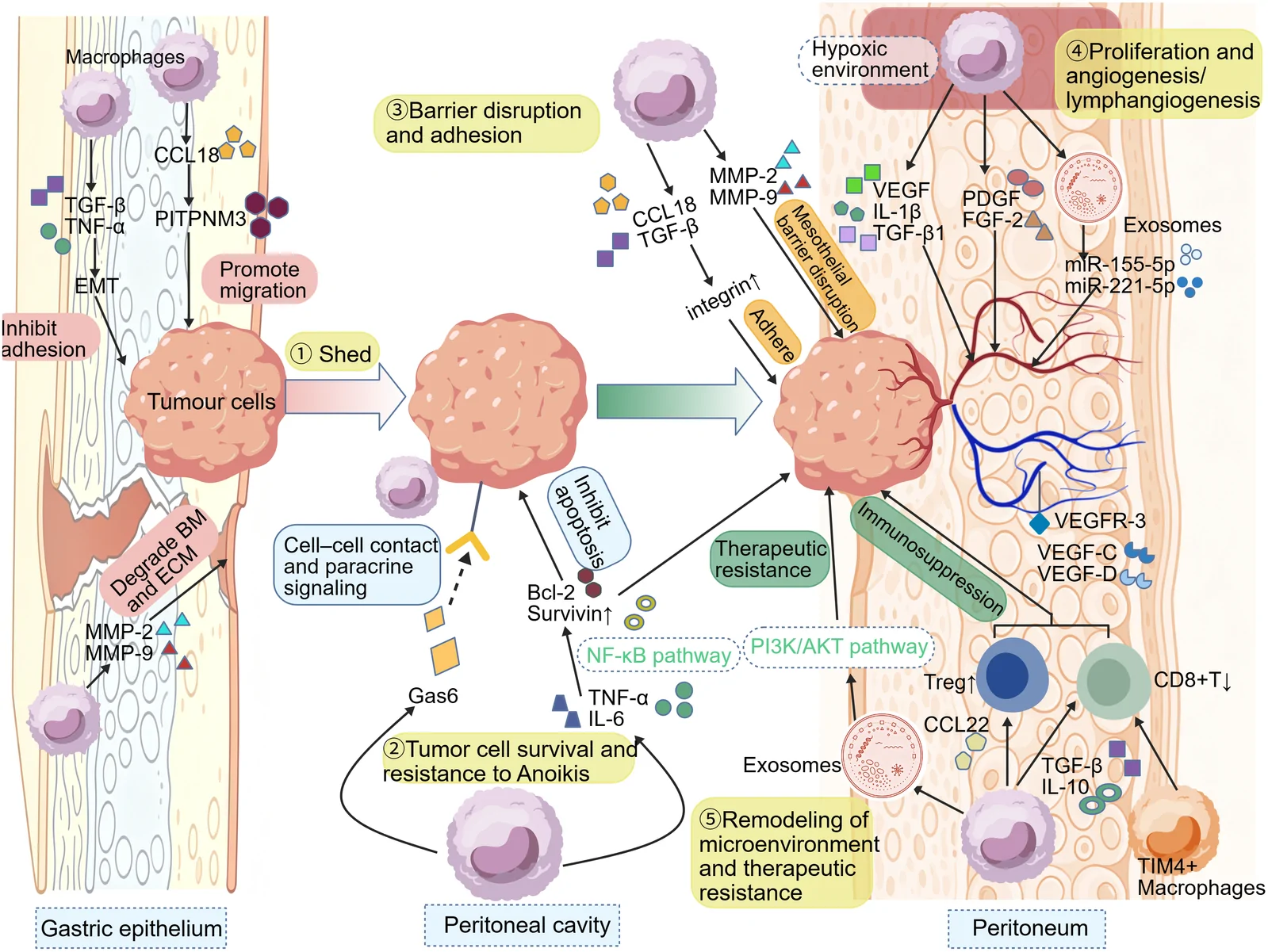
Schematic illustration of macrophage-mediated regulation in gastric cancer peritoneal metastasis. TAMs promote the peritoneal metastatic cascade through five key steps: Tumor cell detachment from the primary gastric lesion via EMT induction (TGF-β, TNF-α) and basement membrane degradation (MMP-2/9, CCL18/PITPNM3); Anoikis resistance through NF-κB and PI3K/AKT pathway activation (IL-6, TNF-α, Gas6), upregulating anti-apoptotic proteins (Bcl-2, Survivin); Mesothelial barrier disruption and adhesion via MMP-mediated ECM degradation and integrin upregulation; Angiogenesis and lymphangiogenesis under hypoxic conditions (VEGF, PDGF, FGF-2, exosomal miRNAs); and Immunosuppressive microenvironment remodeling and therapeutic resistance via cytokine secretion (IL-10, TGF-β), Treg recruitment (CCL22), effector T cell phagocytosis (TIM4^+^), and exosomal signaling. TAM, tumor-associated macrophage; EMT, epithelial–mesenchymal transition; MMP, matrix metalloproteinase; ECM, extracellular matrix; VEGF, vascular endothelial growth factor; PDGF, platelet-derived growth factor; FGF, fibroblast growth factor.

The peritoneal metastasis of gastric cancer follows a multi-step pathological process. When tumor cells detach from the primary stomach, once they enter the abdominal cavity, these cells must first resist apoptosis in a harsh microenvironment before they can break through the mesothelial cell layer, attach and colonize. Then, they will spread to the surface of the peritoneum and grow into visible tumor foci. In this process, angiogenesis and lymphangiogenesis provide nutrition for the tumor and form a further diffusion pathway (37). At the same time, macrophages in the metastatic focus change the local immune balance, promote chemotherapy resistance, accelerate the progression of the disease, and pose a major obstacle to treatment. Given the remarkable plasticity and functional diversity of macrophages, they participate in almost all critical steps in this metastatic process. In the following part, we will discuss how they promote tumor cell shedding, enhance apoptosis resistance, weaken the mesothelial barrier to facilitate adhesion, support the formation of new blood vessels and lymphatic vessels, establish an immunosuppressive environment, and promote drug resistance. From the perspective of the hallmarks of cancer, these macrophage-driven processes not only align with classical hallmarks such as sustained angiogenesis and immune evasion but also intersect with several newly proposed dimensions. In the updated framework, unlocking phenotypic plasticity, non-mutational epigenetic reprogramming, polymorphic microbiomes, and senescent cells have been recognized as emerging hallmarks that contribute to tumor progression (38). Macrophages in peritoneal metastasis are intimately linked to these novel hallmarks: they exhibit remarkable phenotypic plasticity, participate in bidirectional epigenetic reprogramming with tumor cells, interact with the local microbiome, and can adopt a senescence-associated secretory phenotype that shapes the pre-metastatic niche. This expanded conceptual framework provides a useful lens for understanding the multifaceted role of macrophages in gastric cancer peritoneal metastasis. **Figure 2** shows the key steps mediated by these effects and the interaction network centered on macrophages.

### 3.1 Promotion of tumor cell detachment from primary lesions

The separation of cancer cells from the primary gastric tumor marks the beginning of metastasis, which depends on EMT and the enzymatic action of the basal membrane. TAMs accelerate this process by releasing TGF-β and TNF-α, which can activate EMT-induced transcription factors in gastric cancer cells - Snail, Slug and ZEB1 (39, 40). As the adhesion connection between cells weakens, tumor cells are more likely to leave their situs. TAMs also secrete matrix metalloproteinases, especially MMP2 and MMP9, which can degrade the basal membrane and extracellular matrix components (41), thus effectively paving the way for cell detachment. In the tumor stroma, M2 polarized macrophages secrete CCL18, which binds to PITPNM3 receptors on gastric cancer cells, significantly enhancing their migration and aggression, and promoting their escape from the primary focus. Recent studies have shown that Fibronectin (FN1) produced by macrophages can upregulate SDC4 in cancer cells, thus inhibiting the Hippo signaling pathway and significantly accelerating the peritoneal metastasis of gastric cancer (42).

### 3.2 Enhancement of tumor cell survival, Anoikis resistance, and metabolic reprogramming

For gastric cancer cells that have spread to the abdominal cavity, the biggest survival obstacle is apoptosis. TAMs help these cells escape from this programmed cell death in many ways. In the malignant ascites microenvironment, IL-6 and TNF-α secreted by TAMs can activate the NF-κB and STAT3 signaling pathways in tumor cells, thus maintaining their survival and promoting their growth (43, 44). This signal transduction further upregulates the expression of anti-apoptotic proteins such as Bcl-2 and Survivin, so that cancer cells can get stronger anti-apoptotic protection. In addition, TAMs also secrete Gas6; when Gas6 binds to Axl receptors on tumor cells, it will activate the PI3K/Akt cascading reaction (45), providing gastric cancer cells with a way to resist apoptosis. At the same time, cancer cells shed from the primary tumor often enter the abdominal cavity in the form of a cell mass called “spherical structure”. There is evidence that macrophages can invade these cell masses and promote their survival through direct cell contact and paracrine factors. In addition, TAM-derived glial cell line-derived neurotrophic factor (GDNF) regulates autophagy through GFRA1 receptor signaling (46), which helps gastric cancer cells to tolerate metabolic stress and continue to progress (47). A similar mechanism is also believed to play a role in the process of peritoneal metastasis.

In addition to these survival signals, macrophages also contribute to the metabolic reprogramming of gastric cancer cells in the peritoneal microenvironment. Tumor cells often rely on aerobic glycolysis and glutaminolysis to meet their biosynthetic and bioenergetic demands, and TAMs can provide key metabolites—such as lactate, pyruvate, and amino acids—that fuel these pathways. For instance, lactate secreted by macrophages can be taken up by cancer cells and used as an alternative carbon source, supporting their survival under nutrient-deprived conditions (48). Moreover, TAM-derived cytokines can upregulate glycolytic enzymes and transporters in tumor cells, further promoting metabolic adaptation and resistance to metabolic stress (49). This metabolic crosstalk between macrophages and tumor cells represents an emerging mechanism by which TAMs sustain tumor cell viability and enhance peritoneal metastatic outgrowth.

### 3.3 Disruption of the peritoneal mesothelial barrier and promotion of tumor cell adhesion

The mesothelial lining forms a major anatomical barrier, which physically prevents unattached cancer cells from adhesion and colonized. Macrophages fight this process by secreting MMP 2 and MMP 9, which can digest the extracellular matrix that supports mesothelial cells (50). This destruction of the matrix will weaken the barrier structure, leading to tissue damage, and finally exposing the lower basement membrane. The newly exposed area becomes a foothold for gastric cancer cells to easily attach. At the same time, macrophages also release CCL18, TGF-β and other mediators to upregulate the integrin (α5β1 and αvβ3) on the surface of cancer cells and enhance their adhesion to the surface of the mesothelial layer (51). In addition, they also directly help tumor cells adhere to the peritoneum by releasing fibronectin, Osteopontin and other matrix proteins (52).

### 3.4 Facilitation of metastatic nodule proliferation and angiogenesis/lymphangiogenesis

Once tumor cells are successfully attached to the peritoneum, their growth and further proliferation depend on the formation of new blood vessels and lymphatic vessels. Hypoxia in the tumor microenvironment is the main trigger. Under hypoxic conditions, macrophages will release a variety of factors - VEGF, IL-1β, TGF-β1, TNF-α, bFGF and IL-10, which together strongly promote angiogenesis (53). In addition, macrophages also secrete PDGF and FGF-2 (54), which can induce the growth of new blood vessels and deliver essential nutrients to the peritoneal metastasis.

The exosome released by M2 polarized TAMs carries miR-155-5p and miR-221-5p; these tiny RNAs target E2F2, thus promoting angiogenesis (55). Unlike other organ metastasis, the peritoneal spread of gastric cancer is highly dependent on lymphangiogenesis. M2-like TAMs are the main sources of VEGF-C and VEGF-D. These factors combine with VEGFR-3 on lymphatic endothelial cells to stimulate the germination and expansion of lymphatic vessels (54, 56), form drainage channels, and promote tumor proliferation. The newly formed lymphatic network not only provides nutrition for the metastatic focus, but also enables cancer cells to spread systematically (54), thus promoting a vicious circle and exacerbating peritoneal metastasis.

### 3.5 Remodeling the immune microenvironment and inducing chemotherapy resistance

Once tumor cells are colonized on the peritoneum, their survival and proliferation require not only nutrients from new blood vessels, but also to escape immune attacks, which may be more important. The microenvironment of peritoneal metastasis of gastric cancer has a strong immunosuppressive effect, of which macrophage polarization is the core factor leading to this condition. M2-type polarized macrophages inhibit the anti-tumor immune response by releasing IL-10 and TGF-β (57, 58), which directly blocks CD8^+^ T cell activation. TAMs also recruit Tregs via CCL22/CCR4, drive CD8^+^ T cell exhaustion through PD-L1/PD-1 and Galectin-9/TIM-3 (59), and promote MDSC recruitment via CXCL1/2/5, IL-6, PGE2 and S100A8/A9. These suppressive cells further reinforce M2-like polarization of TAMs and blunt chemotherapy-induced apoptosis, thereby promoting drug resistance. TIM4+ macrophages can recognize phosphatidylserine on the surface of CD8+ T cells in apoptotic phase and remove it through phagocytic action (60), which helps to control the anti-tumor immune response. Similarly, TIM3+ TAMs promote gastric cancer progression and peritoneal metastasis by inhibiting T cell-mediated immune response (61). In addition to these immune-mediated mechanisms, macrophages directly promote chemoresistance: M2-polarized TAMs can release IL-6 and TNF-α, activate the NF-κB and STAT3 signaling pathways in gastric cancer cells, and upregulate anti-apoptotic proteins such as Bcl-xL and Survivin (62, 63), thus weakening the response of tumor cells to chemotherapy and promoting drug resistance. Macrophages can also act as a “drug barrier”, clearing chemotherapy drugs through phagocytosis, or physically blocking them from entering the tumor tissue. The exosome released by M2 polarized macrophages further promotes chemotherapy resistance. In a reported mechanism, the miR-21 carried by the exosome of M2 macrophages targets PTEN, activates the PI3K/Akt signaling pathway, inhibits cell apoptosis, and finally makes gastric cancer cells resistant to cisplatin (54, 64).

## 4 Key molecular mechanisms and signaling networks

The functional regulation of macrophages in peritoneal metastasis of gastric cancer is by no means simple. It depends on the joint action of multiple signals from different levels. The polarization and activation of macrophages are determined by the coordination of multiple intracellular signaling pathways, while the downstream effect is amplified and continuously maintained through the signals emitted by neighboring cells. In the next chapter, we will deeply analyze this regulatory system from four aspects: core signaling pathways, intercellular signal networks, complement-mediated lineage transformation, and two-way communication through exosomes.

### 4.1 Core signaling pathways regulating macrophage polarization and function

The activity of macrophages in peritoneal metastasis of gastric cancer depends on the interaction between multiple signaling pathways, one of which has become a key regulatory factor. A basic early step is the recruitment of macrophages - that is, it is necessary to attract macrophages into the abdominal cavity. The CCL2/CCR2 axis plays a major driving role in this process. CCL2 is mainly released by tumor cells and mesothelial cells, and can bind to CCR2 on monocytes and macrophages, thus attracting monocytes in the circulation to the peritoneal cavity (65). In animal models, CCR2 can significantly reduce the infiltration of peritoneal macrophages through gene knockout or drug blocking, which clearly indicates that the pathway provides the necessary upstream basis for TAM generation.

Once macrophages enter the abdominal cavity, they need strong survival and proliferation signals to survive, and the CSF-1/CSF-1R axis just provides this signal. CSF1 (M-CSF) produced by gastric cancer cells and cancer-related fibroblasts (CAFs) can bind to CSF-1R on TAMs, thus activating phosphatidyl inositol 3 kinase (PI3K)/Akt and mitogen-activated protein kinase (MAPK)/extracellular Signal regulation kinase (ERK) pathway (66). This signal is like a lifeline, maintaining the survival of macrophages, promoting their proliferation, and maintaining their differentiation (67). In the peritoneal metastasis model, CSF-1R inhibitors such as pexidartinib can reduce TAM infiltration (68) and significantly delay the expansion of metastatic foci. Clinical observations also support this trend: the abnormal activation of the CSF-1/CSF-1R axis in gastric cancer is closely related to lymph node metastasis and peritoneal dissemination.

Macrophage polarization does not depend on a single trigger, but is determined by the interaction between multiple signaling pathways. STAT3 and STAT6 are the main transcription drivers of M2 polarization. IL-4 and IL-13 combine with IL-4Rα to activate STAT6, thus directly activating M2 characteristic genes, such as Arg1 and CD206. At the same time, IL-6 and IL-10 upregulate M2-related factors through Janus kinase/signal transducer and activator of transcription 3 (JAK/STAT3) signaling pathway (69). Clinically, among the TAMs isolated by malignant ascites in gastric cancer patients, the phosphorylation level of STAT3 and STAT6 is much higher than that of the corresponding cells in peripheral blood, and the degree of phosphorylation is positively related to the high expression level of M2 markers.

The PI3K/Akt pathway is another important participant in the process of regulating polarization. Phosphatidyl inositol 3 kinase γ (PI3Kγ) is widely present in myeloid cells and mediates the polarization of macrophages through the Akt/mTOR axis (70, 71). Using the selective inhibitor IPI-549 to block the pathway can transform M2 polarized macrophages into an anti-tumor state similar to M1, thus helping to restore anti-tumor immune activity (44).

Unlike some signaling pathways, NF-κB does not always act in one direction - its effect depends on the specific environment. In a typical inflammatory environment, the NF-κB signaling pathway promotes the production of pro-inflammatory cytokines. However, the peritoneal metastatic microenvironment has different characteristics, rich in TGF-β and IL-10. This environment is conducive to NF-κB forming p50/p50 homodimers, thus activating genes related to M2, such as IL-10 and CD206 (69, 72). In other words, the actual effect of NF-κB is closely related to its local microenvironment.

These signal channels are not isolated, but intersect and intertwined into a closely connected network. For example, STAT3 can promote the production of CCL2, thus further recruiting more monocytes. At the same time, the PI3K/Akt signaling pathway promotes the phosphorylation of STAT3, thus triggering a self-reinforcing positive feedback loop, so that the signal is constantly amplified (69, 73). Through this multi-level interaction, TAMs can continuously and finely regulate its microenvironment, which is the basis of its high adaptability and flexibility.

### 4.2 Interaction network between macrophages and other microenvironmental cells

In the peritoneal metastasis microenvironment, macrophages do not act in isolation. They are connected to a variety of cell types (mesothelial cells, cancer-related fibroblasts, T lymphocytes and tumor-related neutrophils) through ligand-receptor interactions, forming an interrelated network (74). This cell network promotes peritoneal colonization and promotes tumor progression.

An important result of the interaction between macrophages and mesothelial cells is the destruction of the physical barrier of the peritoneum. Macrophages release matrix metalloproteinase (MMPs), tumor necrosis factor α (TNF-α) and interleukin 1β (IL-1β) (41), thus inducing mesothelial-to-mesenchymal transition (MMT). With the progress of MMT, the markers of mesothelial cells, such as E-cadherin, gradually decrease, while the mesenchymal-like markers increase (51, 75). The destruction of this barrier weakens the mesothelium and provides a channel for cancer cells to invade and colonize the peritoneum.

There is a positive feedback cycle between macrophages and CAFs, which mutually enhance their tumor-promoting function. TGF-β and PDGF released by macrophages can activate stationary fibroblasts and promote their conversion into CAFs (76). In response, these CAFs secrete CSF-1 and IL-6, further tilting macrophages to the M2 phenotype (77, 78). This mutual strengthening continues to promote fibrosis, reshape the extracellular matrix, and promote tumor progression.

Single-cell RNA sequencing adds another meaning to this picture. The interaction between SPP1^+^TAMs and fibroblasts helps to build an immunosuppressive pre-metastasis microenvironment in the peritoneum - this environment is conducive to the survival and growth of tumor cells (79). A recent study reveals that the complement-mediated intercellular communication between CAFs and macrophages is the core mechanism of resistance to immune checkpoint blocking treatment of gastric cancer peritoneal metastasis. Destroying this intercellular communication can reshape the tumor microenvironment and reverse the resistance of immunotherapy (74).

Macrophages also interact with T cells and other immunosuppressive cells to establish a self-reinforcing immunosuppressive microenvironment. As mentioned earlier, macrophages inhibit the activation and function of CD8+ T cells by releasing IL-10 and TGF-β (80). In addition, TAMs recruit CCR4+ Tregs through CCL22, and Treg-derived IL-10 and TGF-β reciprocally reinforce M2-like polarization of TAMs (81). TAMs also express PD-L1 and Galectin-9, which engage PD-1 and TIM-3 on CD8+ T cells and drive terminal exhaustion, characterized by loss of IFN-γ, TNF-α and granzyme B (20, 82); exhausted CD8+ T cells then fail to provide IFN-γ-mediated M1-polarizing signals, consolidating the immunosuppressive circuit. In addition, TAM-derived CXCL1/2/5, IL-6, PGE2 and S100A8/A9 promote the recruitment, expansion and suppressive activity of myeloid-derived suppressor cells (MDSCs) (83). MDSCs in turn produce arginase 1, inducible nitric oxide synthase, IL-10 and TGF-β, which suppress T cells and further shift macrophages toward a pro-tumor phenotype (84). This TAM–Treg–exhausted CD8+ T cell–MDSC network is central to the immune-privileged peritoneal niche.

**Figure 3:**
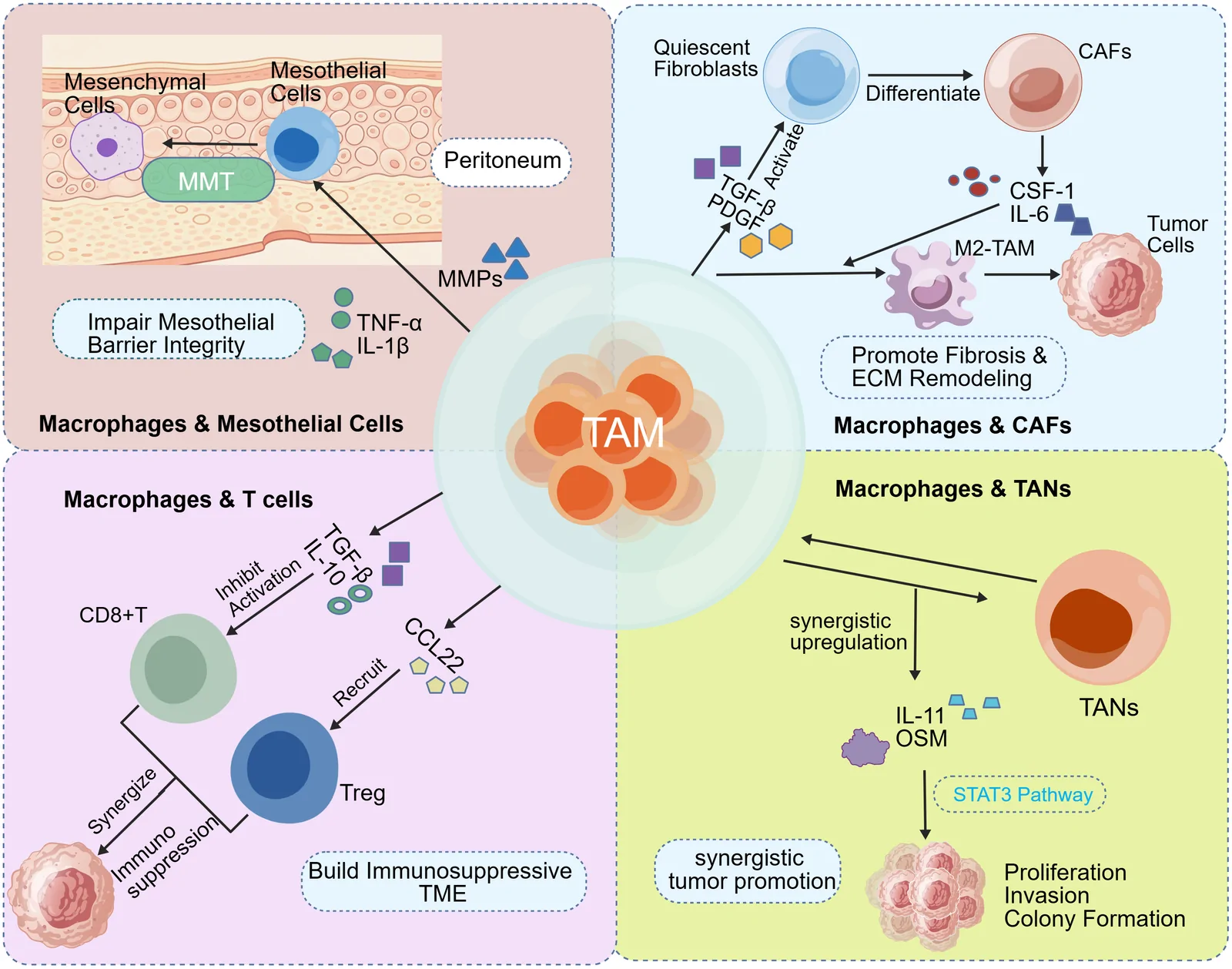
Schematic illustration of the interaction network between macrophages and other cellular components in the peritoneal metastatic microenvironment. Macrophages (TAMs) engage in crosstalk with: Mesothelial cells – secreting MMPs, TNF-α, and IL-1β to trigger mesothelial–mesenchymal transition (MMT), impairing mesothelial barrier integrity; Cancer-associated fibroblasts (CAFs) – macrophages secrete TGF-β and PDGF to activate quiescent fibroblasts into CAFs, while CAFs secrete CSF-1 and IL-6 to further polarize macrophages toward M2-TAMs, forming a positive feedback loop that promotes fibrosis and ECM remodeling; T cells – macrophages inhibit CD8^+^ T cell activation via IL-10, TGF-β, and PD-L1/PD-1, and recruit Tregs via CCL22, collectively building an immunosuppressive TME; Tumor-associated neutrophils (TANs) – synergistic crosstalk leads to upregulation of IL-11 and OSM, which activate the STAT3 pathway in tumor cells, enhancing proliferation, invasion, and colony formation. These interactions collectively foster tumor promotion and immune evasion in peritoneal metastasis.

Macrophages and neutrophils also promote tumor progression through synergy. Co-culture experiments show that the interaction between these two cells will change the spectrum of their cytokines and chemokines, and increase their levels of IL-11 and Oncostatin M (OSM). This in turn activates the STAT3 signaling pathway in tumor cells and enhances their growth, invasion ability and nodule formation ability (85). Therefore, the collaboration between macrophages and neutrophils constitutes an important hub in the network of promoting tumor inflammation in the peritoneal metastasis microenvironment. **Figure 3** summarizes the various ways in which macrophages interact with other cells in the microenvironment.

### 4.3 C1q-mediated complement pathway and TAM lineage switching

Single-cell sequencing technology can now draw a high-resolution map of TAMs heterogeneity. Li and his colleagues conducted single-cell RNA sequencing of ascites samples from 63 gastric cancer patients with peritoneal metastasis, revealing the transformation of TAM subtypes in the process of dynamic changes in ascites (10). Research data shows that TAMs in the ascites continues to transition from the high expression state of CTS to the high expression state of C1q. The first to appear is CTS high TAMs, which secretes CTSS and CTSL, recruits disseminated tumor cells (aDTCs) into the ascites, and then differentiates into C1q high-expressed TAMs. These C1q high TAMs activate the classical complement pathway through C1q, and significantly upregulate PD-L1 and NECTIN2 on disseminated tumor cells in ascites, thus accelerating their proliferation and enhancing immune escape ability.

**Figure 4:**
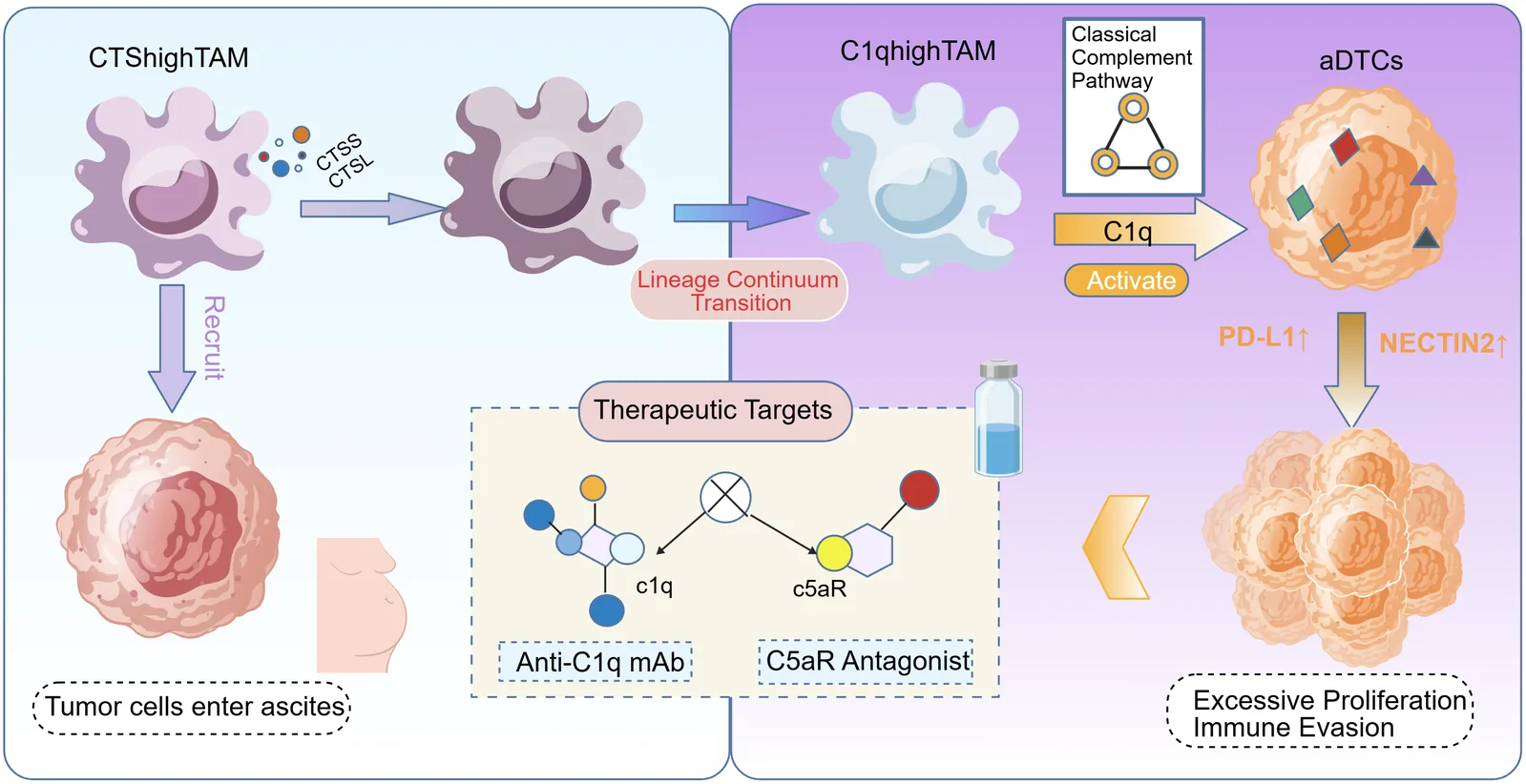
Schematic overview of TAM lineage switching and C1q-mediated complement activation in gastric cancer peritoneal metastasis. Within the ascitic microenvironment, TAMs undergo a continuous lineage transition from a cathepsin-high (CTS-high) phenotype to a complement component 1q-high (C1q-high) phenotype. CTShigh TAMs recruit disseminated tumor cells into ascites via secretion of CTSS and CTSL. Upon transition to C1q-high TAMs, they activate the classical complement pathway through C1q, which in turn upregulates PD-L1 and NECTIN2 expression on ascites-disseminated tumor cells (aDTCs), promoting excessive proliferation and immune evasion. Therapeutic interventions targeting this axis—such as anti-C1q monoclonal antibodies and C5aR antagonists—are shown as potential strategies to block the immunosuppressive cascade.

This study provides a new perspective on how peritoneal metastasis can escape immune control, and highlights C1q high TAMs and downstream complement activation products such as C3a and C5a as potential therapeutic targets. In preclinical studies, anti-C1q monoclonal antibodies and C5aR antagonists have shown the potential to reverse TAM-mediated immunosuppression. However, most of the evidence so far comes from the research of Li and colleagues, and these findings need to be independently verified before they can be widely applied in clinical practice. **Figure 4** summarizes the dynamic transformation process of the TAMs spectrum and the complement pathway driven by C1q, which helps immune escape.

### 4.4 Exosome-mediated intercellular signal communication

As a key messenger, the exosome transmits signals between gastric cancer cells and macrophages (86). For example, miR-21 released by M2 polarized TAMs can target PTEN and activate the PI3K/Akt signaling pathway, thus reducing the sensitivity of gastric cancer cells to cisplatin (64). Tumor-derived exosome can also promote the transformation of macrophages to the M2 state. The exosome released by gastric cancer cells contains circATP8A1, which can bind to miR-1-3p and activate the STAT6 signaling pathway, thus promoting M2 polarization (87). On the contrary, M2 polarized TAMs release the exosome circ_0008253, which can directly upregulate the ATP-binding cassette G subfamily member 2 (ABCG2) and reduce the sensitivity of gastric cancer cells to oxaliplatin (88). MiR-541-5p derived from gastric cancer cells targets dual-specificity phosphatase 3 (DUSP3), activates JAK2/STAT3, and further promotes macrophages into the M2 phenotype (89). In summary, these exosome signals establish a close two-way regulatory loop between tumor cells and macrophages.

TAM-derived exosomes have shown potential as biomarkers in a variety of cancers (90, 91), but their clinical application in gastric cancer peritoneal metastasis still needs to be further studied in depth. Overall, these findings reveal the two-way communication between gastric cancer cells and TAMs driven by exosomes, and this signal network may bring practical potential to combined treatment strategies.

## 5 Therapeutic strategies targeting macrophages

Macrophages play a key role in all stages of peritoneal metastasis in gastric cancer: they help tumor cells detach from the primary focus and resist apoptosis, break through the mesothelial barrier, promote angiogenesis and lymphangiogenesis, establish an immunosuppressive environment, and induce chemotherapy resistance. Therefore, it is not surprising that targeted macrophage treatment has become a very attractive treatment.

**Figure 5:**
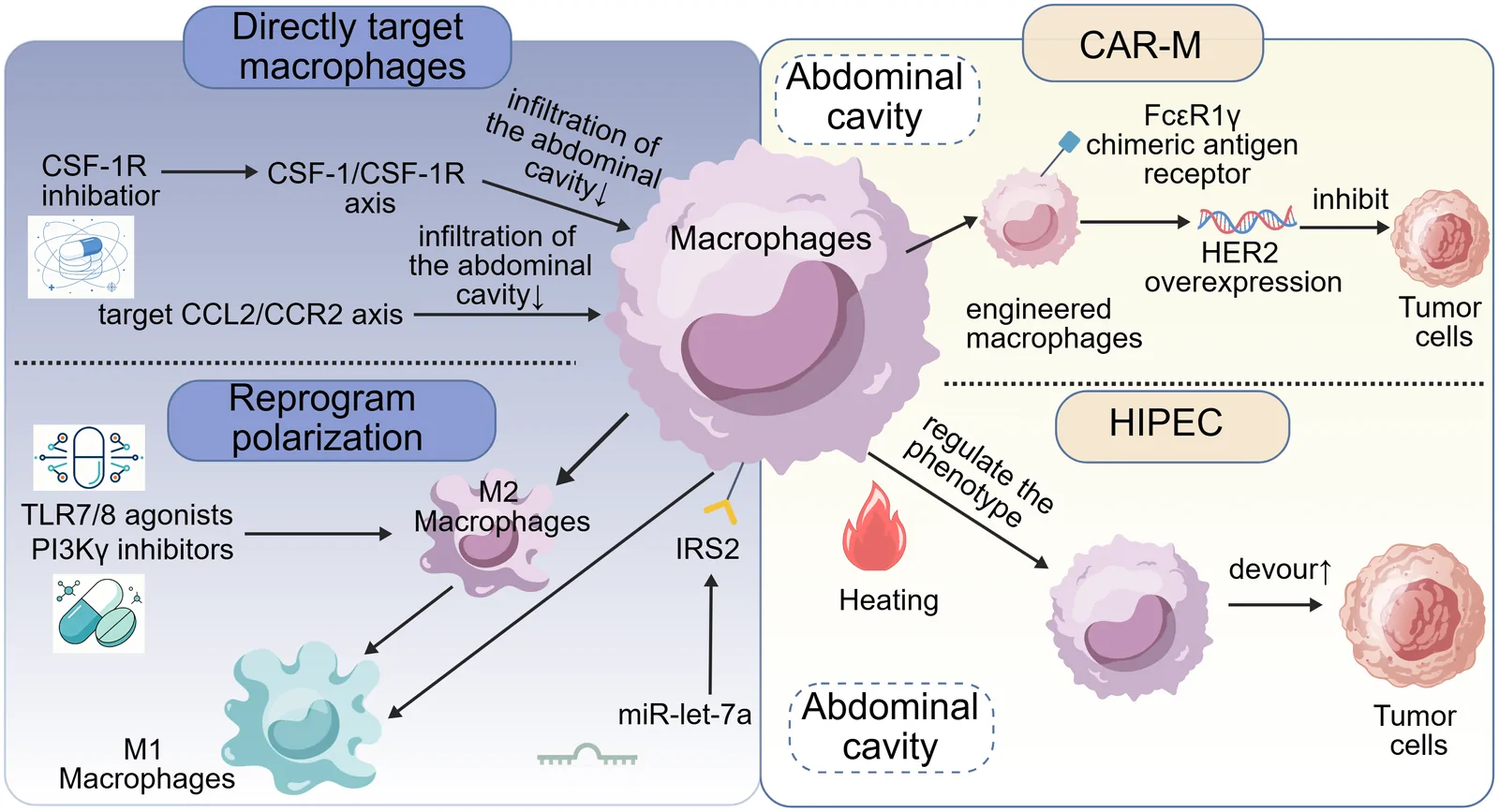
Macrophage-targeted therapeutic strategies for gastric cancer peritoneal metastasis. Schematic overview of macrophage-centered interventions for gastric cancer peritoneal metastasis. Therapies on the left include direct inhibition of macrophage recruitment via blockade of the CSF-1/CSF-1R and CCL2/CCR2 axes, as well as polarization reprogramming using TLR7/8 agonists, PI3Kγ inhibitors, and miR-let-7a targeting IRS2 to shift macrophages from the pro-tumor M2 phenotype toward the anti-tumor M1 phenotype. Treatments applied within the abdominal cavity are displayed on the right: FcϵR1γ-based CAR-M cells inhibit HER2-overexpressing tumor cells, while heating in HIPEC modulates macrophage phenotype and enhances tumor cell phagocytosis.

At present, macrophages-centered strategies can be divided into two categories: one is to directly act on macrophages by blocking their recruitment or changing their polarization state, so that they no longer promote tumor growth; the other is to use cellular and physical methods to activate or enhance the natural anti-tumor ability of macrophages. **Figure 5** provides an overview of these strategies and their potential targets. Next, we will explain these two types of strategies separately.

### 5.1 Inhibition of macrophage recruitment and reprogramming of polarization

One method is to target the CSF-1/CSF-1R or CCL2/CCR2 axis to reduce the infiltration of macrophages into the abdominal cavity (92, 93). CSF-1R inhibitors have been studied in clinical trials for a variety of solid tumors, and the preclinical model of peritoneal metastasis of gastric cancer also shows that it has therapeutic potential (92).

A complementary strategy is to reverse this process and convert tumor-related M2 macrophages into anti-tumor M1 macrophages (94, 95). For example, PI3Kγ inhibitors can effectively reverse M2 polarization and help restore anti-tumor immune function (70). Similarly, Toll-like receptor 7/8 (TLR7/8) agonists can also guide macrophages to M1 phenotype transformation (96).

The nanoparticle delivery system has now created new opportunities for reprogramming macrophages. By encapsulating TLR ligands or targeted small interference RNA in nanocarriers, peritoneal macrophages can be accurately reprogrammed, thus enhancing the therapeutic effect and reducing systemic toxicity. One example is miR-let-7a, which targets insulin receptor substrate 2 (IRS2), promotes macrophages to transform to M1 state, inhibits peritoneal metastasis of gastric cancer, and reshapes the immunosuppressive state in the tumor microenvironment (97).

### 5.2 CAR-macrophages and hyperthermic intraperitoneal chemotherapy

CAR-M is a newer form of cellular immunotherapy. Unlike CAR-T cells, CAR-M can not only directly kill tumor cells, but also reshape the tumor microenvironment, present antigens, and help initiate adaptive immune responses (98–100). In the mouse model, in response to the overexpression of HER2 in the peritoneal metastasis of gastric cancer, the researchers introduced engineered macrophages into the abdominal cavity, and these macrophages were modified to express the FcϵR1γ chimeric antigen receptor (HF CAR-PMs) targeting HER2. The therapy significantly promotes tumor regression and significantly prolongs the overall survival of tumor-bearing mice (101). At present, a clinical trial of HER2-targeted CAR-M treatment for patients with HER2-positive advanced gastric cancer with peritoneal metastasis (NCT06224738) (102) is being conducted. HIPEC is still an important treatment option for gastric cancer accompanied by peritoneal metastasis (103). Part of its therapeutic effect may be mediated by macrophages - only heating can change the phenotype of macrophages and enhance their ability to engulf tumor cells (104).

## 6 Discussion

Although we have made progress in understanding how macrophages promote peritoneal metastasis of gastric cancer, there are still many unknowns. From basic biology to clinical transformation, there are still several key questions to be answered, and the sooner these problems are solved, the more beneficial they will be.

At present, the overall picture of macrophage heterogeneity is still missing. Single-cell RNA sequencing reveals the genealogical transformation of ascitic macrophages from high expression state of CTS to high expression state of C1q, but the key question still exists: do macrophages in different anatomical sites (such as omentum, mesentery and ascites) show different behaviors? Are there any TAM subgroups that have not been discovered yet? Spatial transcriptomics and more advanced single-cell technology are expected to help solve these problems. Recent studies also suggest that CTS+CD68+ macrophages may be potential markers of gastric cancer metastasis (105, 106), and risk models based on M2-type-related genes such as DAB2, SPARC, PLTP and FOLR2 have also been developed (107).

We still know little about the aging of TAMs and how they promote peritoneal metastasis. The latest research shows that aging TAMs actively participates in the formation of metastatic foci during the peritoneal diffusion of gastric cancer. However, how these aging cells reshape the immune landscape in the metastatic microenvironment, or whether they directly induce M2 polarization, needs to be further studied.

Animal models also have obvious defects. The traditional tail vein or intrasplenic injection model cannot truly reproduce the development process of peritoneal metastasis in patients. Therefore, there is an urgent need for a mouse model that is closer to the clinical reality. Organoid co-culture systems and humanized mouse models help to fill this gap.

There are many obstacles to the transformation of macrophage targeted treatment into clinical practice. Since macrophages play an important role in the whole body, systemic targeted treatment may cause serious side effects (7), so it is necessary to use local administration or more selective drugs. In addition, the interaction between TAMs and CAFs can promote the resistance of immune checkpoint inhibitors, which strongly indicates that the combined use of multiple drugs is a reasonable strategy to overcome this obstacle (74).

The combined treatment plan itself still needs to be further optimized. At present, how to combine macrophage targeted therapy with chemotherapy, immunotherapy or HIPEC therapy is still an unsolved mystery. For example, the use of immune checkpoint inhibitors alone has limited effect on peritoneal metastasis of gastric cancer, but if it is used in combination with the treatment of TAMs, it may be significantly enhanced with the help of the reshaped immune microenvironment.
